# Teleconsultation Using Mobile Phones for Diagnosis and Acute Care of Burn Injuries Among Emergency Physicians: Mixed-Methods Study

**DOI:** 10.2196/11076

**Published:** 2018-10-19

**Authors:** Anders Klingberg, Lee Alan Wallis, Marie Hasselberg, Po-Yin Yen, Sara Caroline Fritzell

**Affiliations:** 1 Department of Public Health Sciences Karolinska Institutet Stockholm Sweden; 2 Division of Emergency Medicine Faculty of Medicine and Health Sciences Stellenbosch University Cape Town South Africa; 3 Institute for Informatics, Division of General Medical Sciences Department of Medicine Washington University School of Medicine St. Louis, MO United States; 4 Goldfarb School of Nursing Barnes–Jewish College St. Louis, MO United States

**Keywords:** mobile phone, referral and consultation, emergency medicine, mHealth, teleconsultations, burns, usability evaluation, think-aloud, video analysis, South Africa

## Abstract

**Background:**

The referral process in acute care remains challenging in many areas including burn care. Mobile phone apps designed explicitly for medical referrals and consultations could streamline the referral process by using structured templates and integrating features specific to different specialties. However, as these apps are competing with commercial chat services, usability becomes a crucial factor for successful uptake.

**Objective:**

The aim of this study was to assess the usability of a mobile phone app for remote consultations and referrals of burn injuries.

**Methods:**

A total of 24 emergency doctors and 4 burns consultants were recruited for the study. A mixed-methods approach was used including a usability questionnaire and a think-aloud interview. Think-aloud sessions were video-recorded, and content analysis was undertaken with predefined codes relating to the following 3 themes: ease of use, usefulness of content, and technology-induced errors.

**Results:**

The users perceived the app to be easy to use and useful, but some problems were identified. Issues relating to usability were associated with navigation, such as scrolling and zooming. Users also had problems in understanding the meaning of some icons and terminologies. Sometimes, some users felt limited by predefined options, and they wanted to be able to freely express their clinical findings.

**Conclusions:**

We found that users faced problems mainly with navigation when the app did not work in the same way as the other apps that were frequently used. Our study also resonates with previous findings that when using standardized templates, the systems should also allow the user to express their clinical findings in their own words.

## Introduction

The referral process between primary health care and specialized services remains challenging for several reasons, particularly in resource-constrained settings where specialists are in short supply. Inappropriate or delayed referrals result in inefficient use of resources, both financial and human, but more importantly, it may result in suboptimal care for patients [1].

Electronic referral and consultation systems have been suggested as a promising replacement for paper-based referrals [2,3]. Better medical decisions can be made as all relevant patient and clinical information is available to both the referring clinician and the specialist. Moreover, with electronic systems, the speed of communication and referrals is faster, and the quality of the information exchange is improved by using standardized templates compared with referral letters with illegible handwriting [3].

One area where over- and under-referrals are common is within burn injury care [4-9]. Two core components in the referral process of burn injuries are accurate diagnosis by the initial provider and the ability to effectively communicate these findings to a specialist for referral and management advice. Due to the visual nature of burn wounds, burn care has a tradition of utilizing image- and video-based telemedicine to enhance clinical practice and improve outcomes in patients [10-17]. In the past, telemedicine systems have relied on expensive and bulky infrastructure, which makes implementation difficult in resource-limited settings [18]. Furthermore, burn injuries are still a significant problem, especially in low- and middle-income countries [19]. For best possible outcomes in this group of patients, the decision on management and facility destination (whether to transfer or not) must be made in a timely manner. As burns are often difficult to assess by inexperienced doctors [20-25], remote assistance can be crucial for further management and final disposition. Inaccurate estimation of the size and depth of the burn can lead to over or under fluid resuscitation with adverse effects [26]. Therefore, effective consultation is paramount.

In recent years, as an ad hoc solution to the shortcomings in the referral and consultation process, communication via instant messaging using mobile phones has increased among health care workers. This is exemplified by the increasing reports on the use of chat services such as WhatsApp (WhatsApp Inc) for clinical consultations both within and between hospitals [27-32]. The use of such chat services has often evolved spontaneously from a need for more straightforward and faster channels of communication [33]. Benefits reported include shorter response time [34], flattening of hierarchies, and the ability to break down geographical barriers [35]. Areas in which the use of these chat services are particularly appealing are those with a prominent visual component [31,35], including diagnosis and management of acute burns [32,36]. However, there are some issues using services such as WhatsApp in medical practice. One drawback is that the information that is sent is less structured and often without patient identifiers, making it hard to keep track of which patient is being discussed [37]. In addition, the information including images will not be documented within the hospital information system [37]. Furthermore, despite some authors emphasizing the security of WhatsApp due to its end-to-end encryption [31,32,35,36], patient-related information including images is nonetheless stored on users’ phones, and it is ultimately up to the user to delete messages when no longer needed. Another problem is that by default, WhatsApp saves images and videos to the users’ photo gallery, and depending on the settings, images may be uploaded to other third-party cloud services. On the other hand, deleting messages makes it impossible for information to be audited in the future. There have been attempts to resolve these issues by developing apps intended explicitly for medical consultations [38-42]. Apps such as these can be tailored to contain a premade form to add demographic and clinical information, a chat function, and other features. This approach could allow for smoother consultations as the referring clinician will be prompted to provide the information that the consultant is requesting [3].

Regardless of the app being used, besides proper implementation, it is essential to assess user needs and their perceptions [43]. The most important aspect of technology acceptance and uptake among users is perceived usefulness—“the degree to which a person believes that using a particular system would enhance his/her job performance” [44]. Another critical factor, especially for continuous use, is ease of use—“the degree to which a person believes that using a particular system would be free from effort” [44]. The International Standards Organization (ISO 9241-11) defines usability as the “extent to which a system, product or service can be used by specified users to achieve specified goals with effectiveness, efficiency and satisfaction in a specified context of use” [45].

From our experience in implementing a mobile phone-based referral and consultation system for acute burn injuries in South Africa, uptake has been rather slow, where physicians still choose to call the burns consultant or send them a message via other general text messaging apps (primarily WhatsApp) [32,36]. For example, in a recent report from Cape Town, South Africa, WhatsApp was the preferred method for communicating clinical findings for pediatric burn care [36]. Although there are several reasons for the low uptake of new systems, usability is one important aspect to consider. Therefore, the aim of this study was to assess the usability of a mobile phone app for remote consultations and referrals of burn injuries.

## Methods

### The Vula App

**Figure 1:**
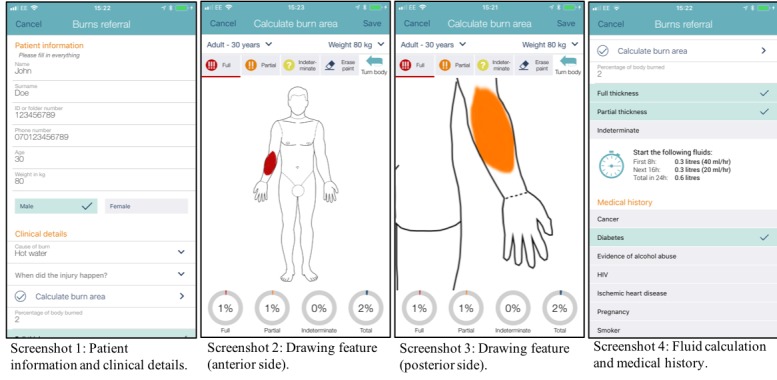
Screenshots of the user interface of the Vula app for burn injury consultations and referrals.

Vula mobile is a mobile phone app for remote consultation and referrals between emergency doctors at point of care and specialists, which was developed by Vula Mobile, Mafami Pty Ltd. The app runs on both iPhone and Android operating systems, and it can be downloaded free of charge. The app was introduced to the Western Cape, South Africa, in 2014 and now allows for referrals to 15 specialties such as ophthalmology, orthopedics, dermatology, and burns. The app currently handles over 5000 referrals per month, with 62 specialist teams actively taking referrals on Vula. The ability to refer patients with burns was introduced in April 2016, and it handled around 250 referrals during 2017. The app provides a template for each specialty, including patient and clinical data (see Figure 1, screenshot 1). There are specific features related to each specialty, the ability to take photographs, and a chat function. The burns section of the app includes a feature to draw the burn on a depiction of a body, which will calculate burn size and fluid requirements (see Figure 1, screenshots 2, 3, and 4). This feature also allows the burns consultant to see the location, size, and depth of the burn. All the documented information including photos are only saved within the app and not saved elsewhere on the phone. The form and the features were established in collaboration with burns experts and emergency specialists before the development of the burns section of the app. When a referring clinician has completed the form, the information is uploaded to a secure server and shared with the burns specialist on call who will be notified. The specialist reviews the information on their mobile phone or computer and sends back treatment and referral advice either via predefined medical advice options or an instant messaging function. After the consultation is complete, the specialist can archive the referral, which will remove it from both the referring doctor’s and the specialist’s devices. Users are mandated to take the necessary precautions in accordance with the “South African Protection of Personal Information Act, 2013” and to act in accordance with national and local legislation. The user is prompted by the app to make sure that the patient has consented to data being stored electronically. As with any consultation, whether it is face-to-face or telephonic, both the referring doctor and the specialist have to make the appropriate medical documentation as mandated by the health care organization where they operate. In this study, participants assessed the app on their own device.

### Participants and Setting

Cape Town has experienced rapid urbanization with a large part of its population living in suboptimal housing that is characterized by crowded living situations, lack of running water, lack of electricity, and houses built out of flammable materials [46]. It has a high burden of burn injuries, with an estimated mortality rate of 7.9 per 100.000 person-years [47]. In Cape Town, there are 2 hospitals with specialized burns units serving the Western Cape province. Depending on the burn severity and day-to-day capacity, patients can be treated at local clinics or referring hospitals. Furthermore, to improve referral of patients in the region, referral criteria have been implemented [48]. However, studies indicate that inappropriate or delayed referrals are still commonplace in this region [6].

This study was conducted in the emergency centers at 2 health facilities, 1 district hospital, and 1 health clinic in Cape Town. Participants were doctors sampled at the emergency center at Khayelitsha Hospital and Gugulethu Community Health Centre by convenience sampling. The primary focus of this study is on emergency doctors; however, an additional 4 of the burns consultants who were registered with the app were selected by convenience sampling and interviewed for the study to explore their view of the app. Although training is available upon request, no training is required to use the app. All participants had used the app for other specialties, and some participants used the app for burns consultations. Among the participants, 6 had never used the app for burns, 14 had used it less than 5 times, and 4 had used it more than 5 times. The burns consultants had all used the app for consultations at least 10 times before the interview.

### Evaluation Methods

This study was a mixed-methods study including a qualitative approach using the think-aloud method and a questionnaire measuring different usability metrics. As the app is already in use, the goal of this usability study was to identify usability problems related to the Vula app to improve the existing app.

#### Think-Aloud Protocol

**Figure 2:**
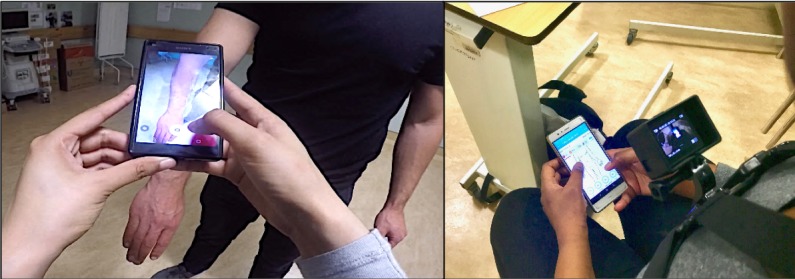
User taking a photo of burn injury (left picture), and camera mounted to user’s chest (right picture).

A think-aloud test was conducted with each participant to assess the usability of the app. The purpose of think-aloud protocols is to have users *think aloud* while performing a set of tasks. The users are asked to verbalize whatever they are looking at, thinking, doing, and feeling [49]. The think-aloud method is suitable to generate data on the cognitive processes when performing a set of tasks [49]. Participants were given a case description of a patient with 2 burn injuries on the right forearm, 1 on the back (posterior side), and 1 on the front (anterior side) (see Textbox 1). Each burn was equal in size and covered about 3% of the body surface, 1 full thickness and 1 partial thickness. The interviewer who acted as the patient had drawn on the arm with a marker to indicate location, size, and depth to facilitate an examination. The participants were asked to use the app as they would in a real situation. Descriptions of tasks for emergency doctors are shown in Table 1 and for burns specialist in Table 2. A camera (GoPro Hero4, GoPro, Inc, San Mateo, California) was mounted to each participant’s chest to record their hands holding the mobile phone (see Figure 2). The interviews took place during their working hours in either an empty examination room or a break room. Interviews lasted for 10 to 20 min. Data collection took place during December 2016 and August 2017. During the second period of data collection, we extended the interview by going through the app once again where the interviewer asked about their thoughts on the different parts and asked further questions about specific problems encountered.

#### Questionnaire

At the end of the interview, the participants completed the previously validated Health Information Technology Usability Evaluation Scale (Health-ITUES) [50]. The Health-ITUES is a customizable questionnaire that subjectively measures the usability of eHealth tools. Each question can be customized to address the specific type of eHealth tool, the type of user, and the specific tasks that users are expected to perform using the system in a specified context. In this study, user=*emergency staff*, *tool*=app, *task*=management of burns, and *context*=emergency care services. The questionnaire covers 4 different domains: quality of work life, perceived usefulness, perceived ease of use, and user control. In total, the questionnaire contains 20 questions measured on a 5-point Likert-scale, ranging from strongly disagree (1) to strongly agree (5), as well as a *non-applicable* option.

**Case description of patient.**

Weight: 80 kg

Sex: Male

Cause of burn: Spilled hot coffee (3% of the body surface, 1 full thickness and 1 partial thickness)

When did the injury happen: 3 hours ago

Medical history: Diabetes; Tuberculosis

**Table 1:** Tasks associated with the Vula app for referral doctors.

Task identification	Tasks associated with the Vula app for referring doctors
Task 1	Launch app from home screen
Task 2	Choose new referral
Task 3	Choose “Burns” in the list of specialties
Task 4a	Choose doctor on call at the referral facility
Task 4b	For first time users: Press the button saying “Yes, I am allowed to refer”
Task 5	Fill out patient information
Task 6	Choose cause of burn from list
Task 7	Select how long ago the burn injury happened
Task 8	Click on “Calculate burn area”
Task 9	On the “Calculate burn area” page, draw the burn on the picture and click save
Task 10	A box will appear with the fluid calculation, and percentage of the burn area will be displayed as well as burn depth.
Task 11	If applicable, select any of the conditions in the list under medical history
Task 12	Take one or more photos of the burn injury
Task 13	Add comments
Task 14	“Click refer” or “Save and refer later”

**Table 2:** Tasks associated with the Vula app for burns specialists.

Task identification	Tasks associated with the Vula app for the specialists
Task 1	Launch app from home screen
Task 2	Select the new referral from the list of referrals
Task 3	Review the information about the referral
Task 4	Go into “Send advice” page
Task 5	Select where the patient should be treated from the drop-down list
Task 6	Choose fluid resuscitation protocol
Task 7	Choose drugs to be given
Task 8	Choose recommended dressings
Task 9	Write further instructions if any
Task 10	Write your assessment of the total burn surface area
Task 11	Write your assessment of the burn depth
Task 12	Click “send advice” button (the user will be taken to the chat window where the selected information has been compiled into a message.)
Task 13	If necessary, chat with the referring doctor

**Table 3:** Usability themes and definitions.

Usability themes	Definition
Usability-related aspects	These codes are used to describe usability problems and issues identified when analyzing video usability data. The codes focus on aspects of the user interface and the user system
Usefulness of content codes	These codes are used to describe issues regarding the usefulness of the user interface or system being evaluated from analyzing the data
Safety- and technology-induced error codes	These codes are used to identify and tag errors made by users when analyzing data

### Data Analysis

Video recordings were analyzed with MAXQDA (Version 12, VERBI Software, Berlin, Germany), a software for analysis of qualitative data. The program allows for analysis of both video and text. First, the audio recordings from the video were transcribed verbatim. In MAXQDA, this can be done using timestamps to have easy access to each audio and video segment that correlates with the text segment in the transcript. The videos can be analyzed by coding directly into the timeline when viewing the video. Content analysis with predefined codes developed by Kushniruk and Borycki was used for coding the data [51]. The codes cover 3 themes: usability, usefulness, and safety- and technology-induced errors (see Table 3). The coding scheme was adapted to suit this study, and codes were also added if findings did not fit the predefined codes. Conversely, some codes were omitted if they did not apply to the material. AK and SCF independently coded all interviews and discussed the labeling of each coded event. PYY worked in the final stage with AK to ensure the codes were accurately assigned to each scenario. The data from the questionnaire were analyzed using descriptive statistics with SPSS (Version 23, IBM Corp, Armonk, NY).

### Ethical Considerations

All participants were presented with both verbal and written information about the study before the interview, and written consent was obtained. Consent forms with participant identifiers were separated from the collected data. Data are presented on an aggregated level. The study was approved by the University of Cape Town, Faculty of Health Sciences Human Research Ethics Committee.

## Results

### Characteristics of Study Participants

Overall, 24 emergency doctors were included in the study, and all of them were working in the emergency department at the 2 different health facilities. Demographics of the study participants are presented in Table 4. Most participants were rotating, that is, they were on a short-term allocation to the emergency center. Experience in emergency care varied from 3 months to 7 years with a median time of 1 year. Participants rated their experience with burn care as either minimal (n=11), moderate (n=10), extensive (n=1), or none (n=2). The participants all used mobile phones for private and work purposes. The purpose of use was sending images, apps, or browsing the internet for reference on medical conditions, criteria, and drug dosage calculations. The mobile phones were also used to discuss cases with seniors using either instant messaging or voice calls.

### Findings From Think-Aloud Sessions

All participants were able to complete all tasks with no major difficulties. However, several usability issues were identified. Usability issues were classified as either *usability problems*, *usefulness of content*, or *safety- or technology-induced errors*, as described in the Methods section (Table 3). Codes and frequency of problems encountered related to each domain in this analysis are presented in Table 5.

#### Usability-Related Aspects

The video analysis revealed 149 problems related to the usability of the app. Most of these problems were related to navigation, consistency, meaning of icons and terminology, and a lack of user instructions. The majority of the problems occurred in the section where the user is prompted to draw the burn on a depiction of a body (Table 1, Task 9). In this part of the app, the most prevalent problems were related to navigation, specifically to zooming and moving the picture.

**Table 4:** Demographics of study participants (N=24).

Characteristics		Participants
**Gender, n (%)**		
	Male	12 (50)
	Female	12 (50)
**Age (in years)**		
	Median	27
	Mean	27.63
	Range	25-34
**Experience in emergency care (in months)**		
	Median (SD)	12 (18)
	Range	3-84
**Experience in burn care, n (%)**		
	None	2 (8)
	Minimal	10 (41)
	Moderate	11 (45)
	Extensive	1 (4)
**Operating system**		
	iOS	15
	Android	9

**Table 5:** Usability codes, definitions, frequency of problems, and number of users experiencing problems.

Code		Definition of code	Times usability problem occurred, n	Users experiencing problems (N=24), n (%)
**Usability codes**				
	Consistency	Relates to aspects of consistency in the user interface	23	10 (42)
	Font	Relates to aspects of font size or text readability	1	1 (4)
	Graphics	Relates to aspects of graphics of the system	4	3 (13)
	Lack of user instructions^a^	Relates to aspects of lack of user instructions	17	10 (42)
	Layout	Relates to aspects of the layout of screens or information on those screens	6	5 (21)
	Meaning of icons/terminology	Relates to aspects of understanding language or labels used in the interface	20	12 (50)
	Navigation	Relates to aspects of moving through a system or user interface	30	14 (58)
	Overall ease of use	Coded when the user makes comments of the overall ease of use of the system	27	9 (38)
	Speed/response time	Relates to aspects of system speed or response time	1	1 (4)
	Understanding instructions	Relates to aspects of understanding user instructions	5	5 (21)
	Visibility of system status	Relates to aspects of understanding what the system is doing	15	11 (46)
**Usefulness of content codes**				
	Accuracy/correctness	Relates to aspects of the accuracy or correctness of information or advice provided by the system	19	14 (58)
	Overall usefulness^a^	Coded when a user makes comments on the overall usefulness of the system	5	3 (13)
	Relevance	Relates to aspects of the relevance of information and features to the user carrying out their task.	30	13 (54)
**Safety- and technology-induced error codes**				
	Mistake	Coded when a review of the data indicates the user has made a mistake that is not corrected	7	4 (17)
	Slip	Coded when a review of the video data indicates the user has made a mistake but corrects the mistake	34	20 (83)
	Work-around	Coded when the user is not using the approach to carrying out work that is recommended by the health care organization or computer system	8	8 (33)

^a^New code added to the original coding scheme.

**Figure 3:**
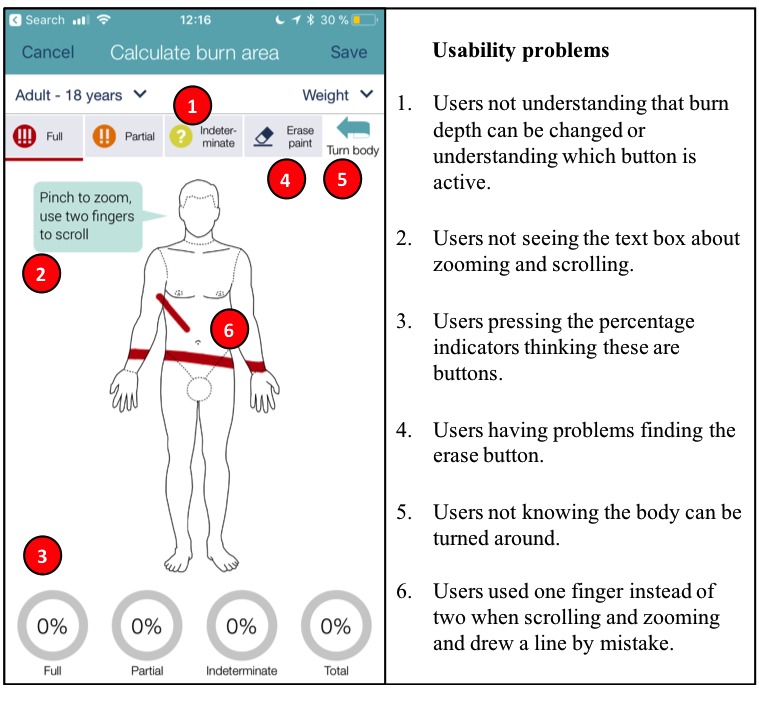
Usability problems identified in the drawing section.

The users often tried to move the picture using one finger instead of two, which resulted in accidental marks which had to be erased (Figure 3, point 6). The users expressed varying degrees of frustration when this happened. A few users mentioned that they did not see the textbox saying “Pinch to zoom, use two fingers to scroll” (Figure 3, point 2), which is related to the codes *lack of user instructions* and *understanding of user instructions*. This textbox is only visible when entering the page and disappears when the user starts drawing. Even if they said they saw it and knew they had to use two fingers to scroll, they would still use one finger out of habit:

So initially it took me a while. Just navigating was a bit hard, because I want to go there, want to move up, and then I kind of you know want to move. I know that you need to put two fingers I’m just not used to it.

User 17

Other problems in this section were related to the layout and meaning of icons and terminology. Some users did not see the erase button and as a work-around, they canceled and re-entered (Figure 3, point 4). A few users also did not see the button for turning the body around to display the back of the patient, resulting in the user drawing both burns on the same side (Figure 3, point 5):

And then also didn’t initially see that there is a turn body to do the other side.

User 17

Similarly, a few users did not know that it was possible to change the color to indicate different burn depths, indicating a lack of user instructions. There was also a problem with the system visibility in this part illustrated by the fact that users tried to press the buttons for burn depth even though they were already selected, which is indicated by a line right beneath the button (Figure 3, point 1). Some users also thought the percentage indicators at the bottom of the screen were buttons to select burn depth (Figure 3, point 3):

I didn’t see that you could change the color of the paint, I just used to write it in the description.

User 22

Some users were also confused about the calculation of the burn surface, and some users did not agree with the calculated percentage in relation to their perception of how big the burn was:

It’s quite difficult to mark with like a finger, because I think with what I’ve drawn and what you have [on your arm] it’s quite a difference, in that it looks like you have a bigger burn on the diagram than what you have.

User 22

Overall, most users found the drawing function to be a useful way to convey the information about burn surface and depth. However, a few users did not find it easy to use and suggested other ways of conveying the burn surface area:

This is too difficult in my opinion because when you try to zoom and then suddenly it draws I’m not a big fan of this I’m not going to lie, I rather just work it out by myself [laughing] than use this.

User 21

#### Usefulness of Content

Many users had comments or experienced issues that related to the content of the app. These were either related to the relevance or accuracy or correctness. One re-occurring theme was that they often could not provide information that was asked for in the app, such as patient’s phone number, weight, or time of injury:

...time of injury is obviously very very important, which I think also gets guesstimated quite a bit here in this area.

User 13

The users did not indicate that any information asked for in the app was redundant. Instead, they suggested more fields or options to provide information in some areas that they thought would be relevant. For example, a section to provide the overall condition of the patient, such as other trauma, or a section where they could describe what management had already been undertaken:

I would rather like this part again to rather to be, type in because this is like limited choice.

User 16

I don’t think there was a section that says what your management has been so far. I think that is quite helpful just to kind of let the other doctor know what you have done.

User 17

Some issues were related to the accuracy and correctness of some of the information. For example, some users had problems selecting the cause of the burn and were debating whether hot coffee should be classified as a hot liquid or as hot water. Although most users did not seem to struggle when choosing the cause, some discrepancy remains; while 8 users chose *hot water*, 16 chose *hot liquid*:

...ok cause of burn, I would choose hot water burn, coffee is basically hot water, but I find that a bit ambiguous

User 2

In addition, some users thought that the list of comorbidities was too limited. Many users suggested that there should be a free text field below each of these sections where they could provide more information.

After the user has finished and saved the drawing of the burn, a box with the fluid calculation is displayed. The users thought this was a useful feature, but some users noted that this was not relevant information, as they would not give intravenous fluid to a patient with a small burn like in the test scenario:

So, the one thing it doesn’t speak about is whether you can use, so a minor burn like that I wouldn’t necessarily go with IV-fluids.

User 12

Many users also talked about how the ability to send pictures was one of the most relevant parts of the app. Most users did not encounter any problems when taking the photos. However, one user thought that the app would automatically save photos to the phone library and was confused when he could not find them. Another user chose to take photos before starting the app and then import the pictures:

I’m going to add the photos from the gallery, because I have the photos already, ehm, so that’s the, where is the picture now? Ok, seems I lost the images.

User 1

At the bottom of the form, there is a field for additional comments, which many users used to write a message to the consultant with information they thought would be relevant. These messages often included information that was already filled in, such as age and gender of the patient or the size of the burn. Many users also used this field to specify that the burn was the result of hot coffee.

#### Safety- and Technology-Induced Error Codes

This theme included slips, mistakes, and work-arounds. One of the most common issues was that users made a slip while using the drawing section. Although this did not cause any significant problems, the users found it frustrating. Only a few users made mistakes that they seemed unaware of making. One user selected an expert from the list that was not on call; during a real scenario, this could have resulted in no response. When using the drawing tool, one user only circled the burn without coloring it in, which resulted in a 0% calculation. The user manually typed in the percentage that the user estimated to be 9%. As some users did not know the body could be flipped around, these users drew both burns on the same side of the body, also resulting in a smaller burn area calculation. Slips, mistakes, and work-arounds were most of the time the result of an underlying usability problem such as understanding user instructions, meaning of icons or terminology, or visibility of system status.

#### Other Insights

During the think-aloud sessions, some users made comments about the app that was not specifically related to the design or functions of the app itself. For example, some users said they would not use the app on a small burn such as the one in the test scenario, that is, they did not find the app useful for smaller burns:

Since it’s not a circumferential burn, I would consider admitting this patient to our surgical team and for them to debride the patient rather than referring the patient. So, I wouldn’t refer the patient via the app.

User 24

Furthermore, when talking about their previous experience using the app, some users said that the specialists were slow to respond, and sometimes they would call them if they had not heard back from them in a while.

### User Satisfaction

Questions and scores relating to each construct in the Health-ITUES questionnaire are presented in Table 6. In general, the app scored high on most of the constructs, with the construct “ease of use” scoring the highest. Usefulness also scored high; however, the questions relating to the ability to receive management advice in a timely manner tended to be lower (items 5, 10, and 12). The construct “user control” scored the lowest, especially the items relating to error prevention (item 18 and 19).

### Interviews With Burns Consultants

A total of 4 interviews were also conducted with burns consultants who used the app to give diagnostic support to emergency doctors. The first screen the consultants see displays all information about the patient sent by the referring doctor. From this page, they can either go into “Send advice,” “Chat,” or review photos. Some consultants did not use the app in the way it was intended to be used; mostly they found it easier to initiate a chat with the referring doctor. In one case, the consultant was unaware of the send advice function. One of the participants said, in general, it is good with predefined options, but in this app, the options were not very useful:

It irritates me that I need to tell them the dose. So, if I choose morphine, I have to write the dose. That’s standard protocol, the nurse or doctor should know this, or use another app, or it should be in this app.

Consultant 1

They all found the pictures to be very beneficial as they could then more accurately assess the burn depth and size. However, 1 consultant expressed that many doctors will not clean the burns or remove blisters before taking the pictures, which then made it harder for the consultant to be able to assess the burn. The consultant suggested there should be instructions in the app about this before the user takes a picture. One consultant seemed unaware that the referring doctor could take pictures within the app and would usually ask them to send pictures through WhatsApp instead.

**Table 6:** Health-Information Technology Usability Evaluation Scale (Health-ITUES).

Item		Concept	Score (1-5)	Cronbach alpha
**Quality of work life**			4.42	.76
	1. I think the app has improved the emergency staff’s ability to care for burns	System impact – career mission	4.67	—^a^
	2. I think the app has been a positive addition to burn care at the hospital	System impact – organizational level	4.46	—
	3. The app is an important part in the acute management of burns	System impact – personal level	4.13	—
**Perceived usefulness**			4.14	.92
	4. Using the app makes it easier to receive expert advice on management of burns	Productiveness	4.5	—
	5. Using the app enables me to receive burn management advice more quickly	Productiveness	3.70	—
	6. Using the app makes it more likely that I have sufficient knowledge on how to manage acute burns	Productiveness	4.00	—
	7. Using the app is useful for receiving information about burn management	General usefulness	4.13	—
	8. I think that the app presents a more equitable process for burn management	General usefulness	4.38	—
	9. I am satisfied with the app for receiving information on burn management	General satisfaction	4.13	—
	10. I can receive information on burn management in a timely manner because of the app	Performance speed	3.88	—
	11. Using the app increases receiving information about burn management	Productiveness	4.33	—
	12. I am able to receive advice on burn management whenever I use the app	Information needs	3.88	—
**Perceived ease of use**			4.64	.74
	13. I am comfortable with my ability to use the app	Competency	4.71	—
	14. Learning to operate the app is easy for me	Learnability	4.63	—
	15. It is easy for me to become skillful at using the app	Competency	4.67	—
	16. I find the app easy to use	Ease of use	4.54	—
	17. I can always remember how to log on and use the app	Memorability	4.67	—
**User control**			3.73	.55
	18. The app gives me error messages that clearly tell me how to fix problems	Error prevention	2.67	—
	19. Whenever I make a mistake using the app, I recover easily and quickly	Error prevention	3.87	—
	20. The information (such as on-screen messages and other documentation) provided with the app is clear.	Information needs	4.33	—

^a^Not applicable.

## Discussion

### Principal Findings

Although the Health-ITUES questionnaire showed the usability to be satisfactory, the think-aloud evaluation revealed several important usability problems that should be considered for improvements for this particular app and for others planning to design apps for remote consultations.

### Usability

Most issues occurred in the drawing feature, which is a central feature of the app that allows the users to describe the size, depth, and location of the burn. For the burns consultant, this information is of great importance when assessing a burn. Some users expressed frustration, and a few even said they thought this feature was too difficult to use. Conversely, many users said that this was one of the strengths of the app, suggesting it may be a central feature that is not present in other communication apps. The consultants also mentioned that being able to see the location of the burn visually is very important for them to do their assessment. This resonates with the findings by Blom et al in their study on expectations of burn specialists about image-based teleconsultation [52].

Given that the drawing function is one of the central features but at the same time where most users struggled, it calls for some reflection. Any extra added feature needs to be well justified, that is, increase the usefulness of the app, but at the same time, it needs to be easy to use, that is, free of effort. Rust et al argue that adding extra features may be attractive but could lead to a product that is overly complex with decreased usability, resulting in *feature fatigue* [53]. Considering these problems that the users encountered, we suggest that any extra feature be well justified (add value to the user) and be designed to be easy to use.

Other usability problems were related to the meaning of icons and their functionalities. Although design changes can improve these, it also demonstrates the importance of user testing with the intended users. Ehrler et al, who found similar issues assessing a mobile app to support nurses, suggest both design changes and also training to mitigate some of these problems [54].

Another finding related to the drawing feature was that some users did not agree with the calculation of the burn surface and said that they would have assessed the burn to be larger had they not used the app. It is well documented in the literature that nonburns specialists tend to overestimate burn surface area when visually assessing the burn [23,24]. One way to at least make it more apparent in the app would be to have the percentage indicated on each body part as a reminder of the burn surface area.

One critical part of usability testing of medical apps is to identify problems that could result in harm to the patient; for example, the fact that some users made mistakes that led to either a smaller or larger calculation of the percentage, which in turn could lead to adverse effects if this discrepancy is significant. This is one of the reasons why supplementing with photos is important so that the burns consultant can make their assessment of the burn.

### Usefulness of Content

Although it is important that new technologies are easy to use, the usefulness of the content of a system is equally or even more important to end users [55]. Although the participants found the content to be useful in the app, many users thought that the app lacked some information or options. First, many users felt limited by the predefined choices and often wanted to describe more about the injury. For example, the medical conditions that are asked for in the app are limited to a few that are of interest to the burn specialist. However, some users thought this list could have been extended. In a similar vein, many users were also not sure whether hot coffee should be classified as *hot liquid, not water* or *hot water*. Both these options are meant to indicate that the burn was a scald. *Hot liquids, not water* is meant to represent what can be referred to as a dense liquid burn that is caused by liquids such as milk and oil [56]. In contrary to less dense liquids such as water, dense liquids will retain more heat and will adhere to the skin longer because of their higher viscosity. Therefore, such burn injuries may result in deeper burns. Although coffee or tea, for example, is not just water, it still has the same properties, which many users also mentioned. However, as one participant pointed out, if milk is added, this will change. In terms of the usability of the app, this relates to the meaning of icons/terminology, as well as information needs. At best, this may only cause confusion and irritation, but there could be instances where misunderstandings might have implications for the patient.

Despite a field for additional comments at the bottom of the form, some users suggested that there should be a possibility to specify with free text under each subsection when the options were too limiting. We did not further explore why some participants felt like this, but one explanation could be that at this point in the user test, they were unaware of the comment box that is located at the end of the form. A study by Hysong et al of e-referral systems reported similar findings where the primary health practitioners felt constrained by the use of templates and that they were not able to communicate findings clearly [57]. Similarly, in the tests with the burn consultants, some of them said they did not like the predefined options in their present form and would rather use the chat function instead.  Hysong et al, on the other hand, found that specialists thought that more rigid templates with mandatory fields would enhance the quality of the referrals [57]. Other studies have found that feedback from consultants is more consistent and timely when referral templates are used [58,59]. One interesting finding regarding the comment box was that many participants used this to write a message to the specialist repeating much of the information already filled out. These messages would often be of a friendly nature including phrases such as “dear Dr” or “kind regards.” This highlights the fact that a consultation is not merely an exchange of information but also a collaboration that requires personal communication [60].

Our findings indicate that both emergency doctors and consultants wanted more flexibility within the system. Flexible systems can make it easier to transfer information about special cases, reducing the need for additional phone and text communication. However, when designing a system, the need for flexibility must be balanced with the need for its usability. Although it might be tempting to design a system for every scenario to maximize flexibility, one needs to take into account that when the flexibility of a system increases, so does its complexity and consequently its usability. This is often termed the flexibility-usability trade-off [61], and when doing this trade-off, it is important to understand the users’ needs, both present and future. This is, however, not always possible, especially with newer technologies. In addition, as acute burn injuries can manifest in different ways, it is difficult to design a template that will fit the clinical presentation of every single patient. Esquivel et al outlined 10 recommendations for improving the effectiveness of electronic referrals [3]. One recommendation that our findings support is to *design and use standardized electronic referral templates that include both structured and free text fields*. They argue that when designing electronic referral templates, there must be both structured fields to capture required information and free text fields for providers to freely expand on their clinical findings.

The usability tests with the consultants revealed that the 2 more experienced users found the ”Send advice” function cumbersome and would rather just chat with the referring doctor. The 2 less experienced consultants seemed to be unaware of some of the functionalities in the app, such as the “Send advice” function or how to access the photos, suggesting that the user interface may not be clear for new users.

### Significance of Findings

Although the participants in our study perceived the burns section of the app to be useful and easy to use, it is still not used on a regular basis. However, there are other reasons for low uptake that are not related to the usability of the app. These include, but are not limited to, awareness of the app, resistance to new technologies and attitudes among colleagues [43]. Even though traditional means of discussing and referring patients with burns by telephonic consultations and paper-based referrals are still prevalent, recent reports from South Africa have described the use of WhatsApp as a mode of communication for clinical decision support [32,36]. The fact that WhatsApp is very prevalent may be a reason why a specific app for medical referrals is not widely adopted. For the referring doctor seeking advice, the most important aspect will inevitably be to receive advice back from the consultant in a timely manner. Therefore, the consultants have an important role to play as pioneers for apps such as the Vula app. Nikolic et al note in their recent study that the widespread use of WhatsApp may impede the introduction of other communication apps designed for medical consultations [62]. Future research should focus on identifying the barriers to using mobile phone-based referral and consultation systems.

### Methodological Considerations

The methods and equipment for data collection used in this study were simple, low cost, and allowed for quick data collection. None of the participants objected or showed any signs of being uncomfortable wearing the recording equipment. The video coding scheme was useful and covered most aspects. However, we changed the original definitions to cover both positive and negative aspects of the events and not only focus on problems. By doing this, the data generated more findings concerning things that the users liked about the app. We also added some codes that we thought were not covered in the original coding scheme. For example, *Lack of user instructions* was added as an extension to the code *Not understanding user instructions*.

During the think-aloud session, the interviewer tried not to interfere, except for encouraging the interviewee to keep talking. However, we found that the participants would mostly verbalize what they were doing but not what they were thinking and feeling. Consequently, during the second period of data collection, we extended the interview by going through the app once again where the interviewer asked about their thoughts on the different parts of the app.

Although the users rated the app to be easy to use, the think-aloud interviews found several problems related to the ease of use. This is not surprising as the purpose of a usability questionnaire is to assess the users’ overall perception of the app and not identify specific problems. It is, however, useful for comparing different user groups, different designs of the app, or how user perceptions change over time.

As this was a mixed-methods study spanning both emergency burn care and health informatics, we think it was a strength that the authors come from different disciplines, including public health, political science, sociology, emergency medicine, nursing, and health informatics. Another strength of the study was the large number of participants in the user tests. A limitation was the relatively homogenous sample, where all users were young junior doctors. Sonderegger et al found that age affects several usability measures such as speed and accuracy [63]. Cillessen et al studied the user satisfaction of a clinical notes app and only differences by medical specialty but no differences by sex, age, professional experience, or training hours [64]. Nevertheless, our sample does represent the typical user from our study population.

### Conclusions

Mobile referral and communication systems are a relatively new concept that could simplify the consultation and referral process within burn care and other specialties. However, when a new system is competing with other technologies such as instant messaging apps or traditional phone calls, usefulness and ease of use become highly important. Although the Health-ITUES questionnaire showed high satisfaction with the system, the think-aloud interviews provided additional insights for further improvements. The users liked the ability to describe the burns through both the built-in drawing function as well as with photographs. For the burn consultants, the drawings and the photos were considered the most valuable information for their assessment. We also found that users had problems with navigation in the drawing section when the app did not act in the same way as other apps that were frequently used. There was also some confusion regarding terminology and the meaning of buttons and icons. Our study also resonates with previous findings that when using standardized electronic consultation and referral templates, the system should also allow the users to freely express their clinical findings in their own words.

## Abbreviations

Health-ITUES: Health-Information Technology Usability Evaluation Scale
